# Green CE–DAD determination of regulated aromatic amines in textiles through DoE optimization

**DOI:** 10.3389/fchem.2026.1952218

**Published:** 2026-09-09

**Authors:** Giulia Simonetti, Francesca Buiarelli, Veronica Mari, Sara Astolfi, Fabio Candiano, Eugenio Sandrucci, Carmela Riccardi, Donatella Pomata, Patrizia Di Filippo

**Affiliations:** 1 Department of Chemistry “Sapienza” University of Rome, Rome, Italy; 2 Wolfson School of Mechanical, Electrical and Manufacturing Engineering University of Loughborough, Loughborough, United Kingdom; 3 Inail DIT-Via Roberto Ferruzzi, Rome, Italy

**Keywords:** aromatic amines, capillary electrophoresis (CE), design of experiments (DOE), diode array detection (DAD), green analytical chemistry, textile products

## Abstract

**Introduction:**

Aromatic amines released from azo dyes are regulated in textile products under the European REACH framework due to their potential health risks. Their determination remains analytically challenging because of their structural similarity, moderate polarity, and the complexity of textile matrices, which can cause significant matrix effects.

**Methods:**

A green Capillary Electrophoresis method coupled with Diode Array Detection (CE–DAD) was developed for the simultaneous determination of eleven regulated aromatic amines in textile extracts. A Design of Experiments (DoE) approach was employed to systematically optimize the separation conditions, with the aim of maximizing resolution, selectivity, and robustness while minimizing analysis time. The environmental sustainability of the analytical procedure was assessed using the BAGI and Agree metric.

**Results:**

The optimized CE–DAD method showed satisfactory analytical performance when validated using spiked textile extracts, providing adequate recovery, precision, and robustness. The method also demonstrated good applicability to complex textile matrices. The green assessment confirmed the low environmental impact of the proposed analytical procedure.

**Conclusions:**

The developed CE–DAD method represents a sustainable, reliable, and cost-effective analytical strategy for the simultaneous determination and monitoring of regulated aromatic amines in textile products. The integration of DoE-based optimization and green analytical assessment highlights its potential as an environmentally responsible alternative for analysis of complex textile matrices.

## 1 Introduction

Aromatic amines are an important class of compounds widely used as intermediates in the chemical industry and, more importantly, are degradation products of azo dyes, which represent the largest class of synthetic colorants employed in the textile industry. Several aromatic amines have been classified as carcinogenic, mutagenic, and reprotoxic (CMR), raising significant concerns regarding consumer exposure through textile products (Rovira et al., 2025), where azo dyes and pigments may also be used for product coloration.

To protect human health, the European Union has introduced strict regulatory measures under Annex XVII of the REACH European Parliament and Council (2006). Regulation (EC) no. 1907/2006, which restricts the use of azo dyes capable of releasing 22 CMR aromatic amines from textile and leather articles intended for direct and prolonged contact with the skin. A maximum concentration of 30 mg kg^−1^ is established for each regulated amine, making reliable analytical methods essential for regulatory compliance, market surveillance, and consumer safety. This need is further reinforced by the globalized textile supply chain, where a large proportion of textile products are manufactured outside the European Union under different regulatory frameworks (European Commission, 2022). The determination of aromatic amines in textile matrices remains analytically challenging because of the high structural similarity among target compounds, their moderate polarity, and the complex composition of sample extracts. Residual dyes, pigments, additives, printing inks, polymers, and finishing agents may generate significant matrix effects that compromise separation efficiency and quantitative performance. Therefore, analytical methods must provide high selectivity, adequate sensitivity, and robust analytical performance. The reference method for the determination of aromatic amines released from azo dyes in textiles is described in the EN ISO 14362-1:2017 standard (International Organization for Standardization, 2017; Tahara et al., 2024). This method is based on the reductive cleavage of azo dyes, followed by chromatographic determination of the released aromatic amines, typically by gas chromatography–mass spectrometry (GC–MS). Although this procedure is well established for compliance testing, it involves multiple sample preparation steps and, depending on the analytical approach, derivatization of free aromatic amines may be required to improve chromatographic performance. These additional steps increase analysis time and may negatively affect method reproducibility, while introducing analytical artefacts (Pinheiro et al., 2004). These drawbacks have stimulated the search for faster and more sustainable analytical alternatives.

In recent years, high-performance liquid chromatography coupled with tandem mass spectrometry (HPLC–MS/MS) has emerged as a powerful alternative owing to its high sensitivity, selectivity, and the possibility of directly determining aromatic amines without derivatization. Nevertheless, both chromatographic techniques require relatively large volumes of organic solvents and expensive instrumentation, limiting their applicability in laboratories seeking greener analytical workflows, and reducing their sustainability for routine analysis (Crettaz et al., 2020; Sutthivaiyakit et al., 2005; Tölgyesi and Sharma, 2020).

In this context, Capillary Electrophoresis coupled with Diode Array Detection (CE–DAD) represents a practical and sustainable alternative for the determination of aromatic amines (Borrós et al., 1999; do Lago et al., 2017; Sałdan et al., 2022). Aromatic amines are weakly basic compounds that can be efficiently and selectively separated under acidic electrophoretic conditions, while exhibiting characteristic UV absorption suitable for DAD detection. Compared with mass spectrometric techniques, CE–DAD relies on simpler and more affordable instrumentation, making it particularly attractive for quality control and regulatory compliance laboratories, where it provides a practical alternative whenever mass spectrometric detection is unnecessary or unavailable. In addition to its high separation efficiency, CE offers minimal solvent and reagent consumption, reduced waste generation, short analysis times, and low operating costs, making it fully consistent with the principles of Green Analytical Chemistry.

Despite these advantages, to the best of our knowledge, this is the first study describing the simultaneous determination of a representative panel of REACH-regulated aromatic amines in textile extracts by CE–DAD combined with multivariate DoE optimization. Because the primary objective of this study was the development and optimization of a CE–DAD methodology, a representative subset of eleven REACH-regulated aromatic amines was selected as model analytes. These compounds were chosen on the basis of their commercial availability and analytical stability. In addition, they cover a broad range of structural features and electrophoretic behaviours, allowing a comprehensive evaluation of the method performance. Experimental conditions were optimized using a Design of Experiments (DoE) approach to evaluate the combined effects of the main chemical and instrumental parameters on separation efficiency, selectivity, time of analysis and method robustness. Compared with the traditional one-factor-at-a-time approach, DoE enables the simultaneous evaluation of multiple experimental variables and their interactions, leading to a more efficient and statistically robust optimization. The environmental sustainability of the optimized method was further assessed using the Blue Applicability Grade Index (BAGI), providing a quantitative evaluation of its greenness and practical applicability. Finally, the optimized method was validated using spiked textile extracts and successfully applied to commercially available textile products, demonstrating satisfactory analytical performance and confirming its suitability for regulatory monitoring of real textile samples. By integrating multivariate optimization with Green Analytical Chemistry principles, this study provides a practical analytical strategy that combines regulatory relevance, environmental sustainability, and operational simplicity. The results demonstrate the potential of CE–DAD as a robust, cost-effective, and environmentally sustainable alternative for the determination of some regulated aromatic amines in textiles extract, thereby expanding the analytical toolbox available for textile safety assessment.

## 2 Materials and methods

### 2.1 Chemicals and reagents

Eleven aromatic amines were chosen as representatives under Annex XVII of the European REACH Regulation, for the development and validation of the proposed CE–DAD method.

The investigated compounds were purchased from Sigma-Aldrich (Merck KGaA, Darmstadt, Germany) and are listed in Table 1. Individual stock standard solutions (1.25 mg mL^−1^) were prepared by dissolving approximately 2.5 mg (weighed using an analytical balance with a readability of 0.001 mg) of each analyte in 2.0 mL of acetonitrile, using a calibrated 2 mL volumetric flask. Working standard solutions were prepared daily by serial dilution with buffer phosphate/methanol solution to the desired concentration. Stock and working solutions were stored in amber glass vials at 4 °C until use.

**TABLE 1:** Target regulated aromatic amines investigated in this study, with their chemical formula, molecular weight (MW), CAS Registry Number, and acid dissociation constant (pKa).

Compound	Formula	MW (g/mol)	CAS no.	*pK*_*a*_ *
o-Toluidine (2-aminotoluene)	C_7_H_9_N	107.15	95-53-4	4.5
p-Cresidine (6-methoxy-m-toluidine)	C_8_H_11_NO	137.18	120-71-8	4.7
4-Aminobiphenyl (biphenyl-4-ylamine,4-aminobiphenyl xenylamine)	C_12_H_11_N	169.22	92-67-1	4.3
Benzidine	C_12_H_12_N_2_	184.24	92-87-5	4.7
4,4′-Methylenedi-o-toluidine	C_15_H_18_N_2_	226.32	838-88-0	5.2
4-Chloroaniline	C_6_H_6_ClN	127.57	106-47-8	4.0
4,4′-Diaminodiphenylmethane	C_13_H_14_N_2_	198.26	101-77-9	5.3
4,4′-Thiodianiline	C_12_H_12_N_2_S	216.30	139-65-1	4.6
p-Phenylazoaniline (4-Aminoazobenzene)	C_12_H_11_N_3_	197.24	60-09-3	∼4.3
o-Aminoazotoluene	C_14_H_15_N_3_	225.29	97-56-3	∼3.1
4-Chloro-o-toluidine	C_7_H_8_ClN	141.60	95-69-2	3.9

* The reported pKa values of the analytes were collected from PubChem and literature source.

HPLC-grade acetonitrile and methanol were used throughout the study (Romil Ltd. Cambridge, UK). Ultrapure water was obtained from a Milli-Q purification system (Millipore, Burlington, MA, United States). Hydrochloric acid (37%), phosphoric acid, formic acid, sodium hydroxide, aqueous ammonia (30%) were of analytical grade and used without further purification (Carlo Erba, Milan Italy).

Florisil (30–60 mesh; Merck, Darmstadt, Germany), quartz sand (0.3–0.9 mm; Büchi, Flawil, Switzerland), RC Filters 0.22 μm (Phenomenex, California, United States) were used.

### 2.2 CE–DAD instrumentation

Capillary electrophoresis analyses were performed using an Agilent 7100 Capillary Electrophoresis system (Agilent Technologies, Waldbronn, Germany) equipped with a diode array detector (DAD). Data acquisition and instrument control were carried out using Agilent ChemStation software (Agilent Chemstation 2010 version B.04.02 version). Separations were performed in an uncoated fused-silica capillary (Agilent Scientific Instruments Santa Clara California) of 50 μm i.d., 50 cm total length and 40 cm effective length. The capillary cassette incorporated an on-column UV detection window, while cartridge temperature was thermostatically controlled throughout the analysis to ensure migration time reproducibility and minimize Joule heating.

Few nL of samples were introduced hydrodynamically by pressure (50 mpsi pressure for 7 s), using the instrument autosampler. CE-DAD analyses were performed following the optimization procedure described in Section 2.6.

### 2.3 Textile samples

**FIGURE 1:**
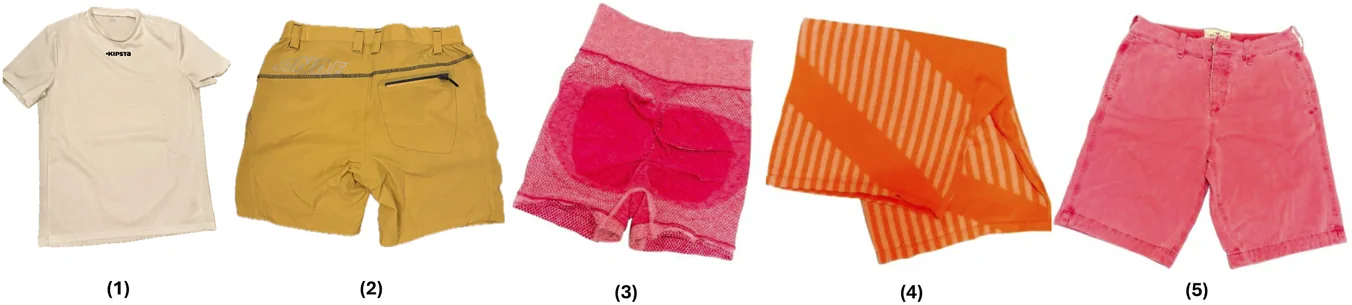
Photographs of the five textile samples investigated in this study. Sample 1 was used as the blank textile sample, while Samples 2–5 correspond to the textile samples analyzed for the presence of regulated aromatic amines.

In this study four items of commercial garments purchased from online fast-fashion stores (samples 2–5) were analysed together with a blank textile sample (sample 1). The samples analysed are shown in Figure 1.

### 2.4 Sample preparation

**FIGURE 2:**
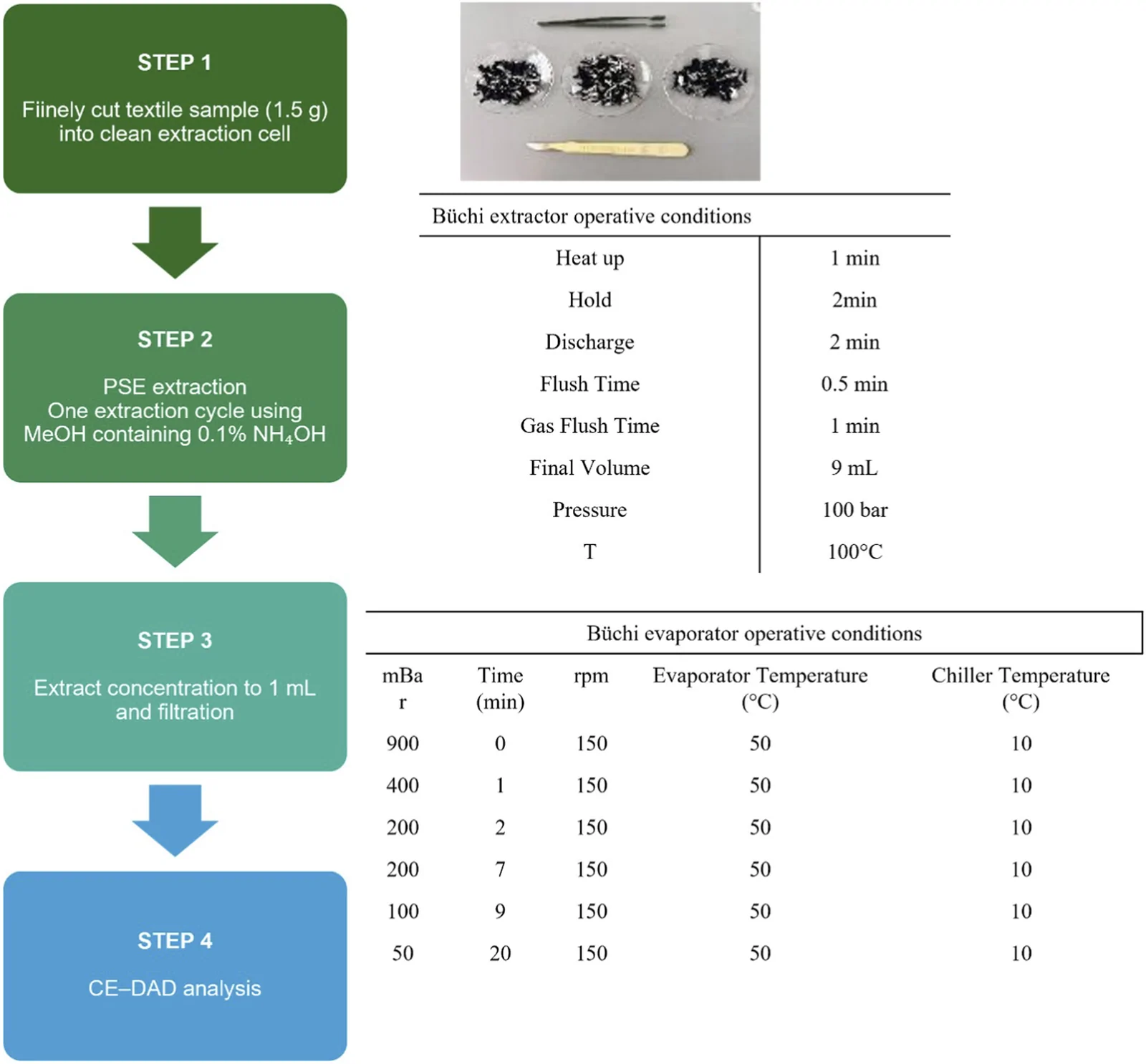
Flowchart of the analytical procedure developed for the determination of regulated aromatic amines in textile samples. The flowchart summarizes the main analytical steps. The extraction conditions are reported in the upper right section of the figure, while the conditions used for solvent evaporation are reported in the lower section.

The overall analytical workflow developed for the determination of aromatic amines in textile samples is summarized in Figure 2. Briefly, 1.5 g of textile sample, finely cut, was extracted by Pressurized Solvent Extraction (PSE), after cell preparation with cellulose filter, frit, quartz sand, and Florisil, using methanol containing 0.1% NH_4_OH. The extract was concentrated to 1 mL, filtered on a 0.22 μm filter, and analysed by CE–DAD.

### 2.5 Method validation procedure

The analytical performance of the developed CE–DAD method was evaluated according to internationally recognized principles for analytical method validation ICH Q2 (R2) guideline (ICH, 2023). Validation was carried out by assessing selectivity, stability, linearity, Detection Limit (DL) and Quantitation Limit (QL), precision, matrix effects, and extraction recovery. Method selectivity was assessed by analyzing blank textile extracts, standard solutions, and spiked textile extracts under the optimized electrophoretic conditions. The absence of interfering peaks at the migration times of the target analytes, together with the evaluation of UV spectra by diode array detection, confirmed the selectivity of the method for the identification and quantification of the eleven aromatic amines in textile extracts. The short-term stability of the working standard solutions was assessed by reanalyzing freshly prepared solutions after 24 h of storage at 4 °C. No significant variations in migration times, peak areas, or electrophoretic profiles were observed, confirming the stability of the analytes under the storage conditions adopted. Calibration curves were obtained by analysing multistandard solutions prepared at six concentration levels ranging from the QL to 60 μg mL^−1^ (equivalent to QL –40 μg g^−1^ in textile matrices). Each calibration level was injected in triplicate under the optimized electrophoretic conditions. Linearity was assessed over the investigated calibration range by ordinary least-squares (OLS) linear regression of the analytical response versus analyte concentration. The regression equation (y = bx + a), slope (b), intercept (a), and coefficient of determination (*R* ^2^) were calculated for each analyte. The uncertainty associated with the regression parameters was expressed as the 95% confidence interval (CI) of the slope and intercept, calculated from their respective standard errors using the Student’s t-distribution with n − 2 degrees of freedom. Residuals were calculated as the difference between the observed and predicted responses and plotted against concentration. The residual plots were visually examined for systematic trends or deviations from the linear regression model over the analytical range investigated. DL values were experimentally established as the lowest concentration providing a signal-to-noise ratio of approximately 3. QL values corresponded to the lowest concentration that provided a signal-to-noise ratio of approximately 10. Precision was assessed by evaluating intra-day repeatability and intermediate precision reproducibility, expressed as relative standard deviation (RSD). Intra-day precision was determined through repeated analyses (n = 10) performed within the same day, whereas intermediate precision was evaluated over three consecutive days by a different operator. The efficiency of the sample preparation procedure was assessed through recovery experiments performed in triplicate on blank textile samples fortified before extraction at the concentration level corresponding to the REACH regulatory limit (30 mg kg^−1^). Recoveries were calculated by comparison with blank textile extracts fortified after extraction. Matrix effects were evaluated by comparing the slopes of calibration curves prepared from blank textile extracts fortified after extraction (matrix-matched calibration curve) with those obtained from standard solutions prepared in phosphate buffer (solvent calibration curve). The matrix effect (ME) was calculated according to Equation 1:

$$ME\;\left(\%\right)=\left[\frac{Slope\;solvent\;}{Slope\;matrix}-1\right]x\;100$$ (1)

Positive values indicate signal enhancement, whereas negative values indicate signal suppression caused by co-extracted matrix components. Matrix effects within ±10% were considered negligible, values between ±10% and ±30% were considered moderate, and values exceeding ±30% were considered significant. Method robustness was evaluated by examining the effect of small deliberate variations in the critical experimental parameters (buffer concentration, applied voltage, and capillary temperature) around the optimized conditions.

### 2.6 Method optimization

The capillary electrophoresis method was optimized through a multivariate Design of Experiments (DoE) approach using MATLAB software. Preliminary screening runs allowed the selection of three independent operational variables to define the experimental domain: buffer concentration, applied voltage, and capillary temperature.

A D-optimal experimental design was selected to maximize the precision of the estimated model coefficients while minimizing the experimental workload. The software generated an experimental matrix consisting of 13 runs (Table 2). In this matrix configuration, rows represent the individual experimental runs (or specific sample conditions), and columns correspond to the independent variables. The system responses were modelled using a second-order polynomial equation to simultaneously evaluate linear effects, two-factor interactions, and quadratic behaviours. Hydrodynamic injection parameters were kept constant at 50 mbar for 7 s across all experiments to ensure reproducible sample loading.

**TABLE 2:** Experimental matrix generated using the D-optimal design for the optimization of the CE-DAD method, showing the experimental levels of buffer concentration, applied voltage, capillary temperature for the 13 experimental runs and the model’s response in terms of number of chromatographic peaks.

No of experiments	mM	kV	°C	No of resolved peaks
1	15	10	25	8
2	30	10	25	10
3	50	10	25	9
4	30	20	25	11
5	50	20	25	10
6	15	30	25	9
7	30	30	25	10
8	50	30	25	8
9	15	10	35	7
10	50	10	35	8
11	30	20	35	10
12	15	30	35	8
13	50	30	35	7

### 2.7 Analytical GREEnness metric approach and blue applicability grade index

The Analytical GREEnness Metric Approach (AGREE), proposed by Pena-Pereira et al. (2020), and the Blue Applicability Grade Index (BAGI), developed by Manousi et al. (2023), are complementary tools for the comprehensive assessment of analytical methods. AGREE evaluates the environmental sustainability of an analytical procedure by integrating the twelve principles of Green Analytical Chemistry (GAC) into a single quantitative score ranging from 0 to 1. The assessment considers key aspects of the analytical workflow, including sample preparation and amount, miniaturization and automation, derivatization, waste generation, throughput, energy consumption, use of renewable resources, hazardous substances, and operator safety. The results are presented as a circular pictogram divided into twelve sectors, providing an immediate visualization of both the individual criteria and the overall greenness score. The weighting of individual criteria can also be adjusted according to the specific analytical context. BAGI, developed within the framework of White Analytical Chemistry (WAC) (Nowak and Kościelniak, 2019; Nowak et al., 2021), complements the environmental assessment by focusing on the practical applicability of an analytical method. The index evaluates ten attributes related to operational efficiency, including the type and number of analytes, sample parallelization, sample preparation complexity, throughput, reagents, preconcentration, automation, and sample amount. Each attribute is assigned a discrete score, contributing to an overall value ranging from 25 to 100, with higher scores indicating greater practical applicability; methods achieving a score ≥60 are considered practical. The results are represented by an asteroid-shaped pictogram, whose colour provides an immediate qualitative indication of applicability. By combining AGREE and BAGI, it is therefore possible to obtain a more comprehensive evaluation of an analytical method, considering both its environmental sustainability and practical applicability.

## 3 Results and discussion

### 3.1 Method optimization via DoE approach and separation/identification performance

The CE-DAD method was optimized using a multivariate Design of Experiments (DoE) strategy. This approach was selected over the conventional one-variable-at-a-time (OVAT) method to evaluate simultaneous interactions between multiple experimental factors, while minimizing the required number of runs.

Electrophoretic separation was performed under acidic conditions using a phosphoric acid buffer (pH 2.5) to ensure complete protonation of the target aromatic amines. Under these strongly acidic conditions, the electroosmotic flow (EOF) was effectively suppressed, rendering analyte migration solely dependent on intrinsic electrophoretic mobility. Because migration behaviour is governed by several interdependent variables, a D-optimal experimental design was employed to identify the critical factors influencing separation efficiency and method robustness. Unlike standard symmetrical designs (such as Central Composite or Box-Behnken), the D-optimal approach is a computer-generated design that selects the best subset of experiments by maximizing the determinant of the information matrix. This mathematically ensures the highest precision in estimating the model coefficients and effectively handles multi-variable spaces with potential experimental constraints.

Preliminary screening experiments were conducted to define the experimental domain. Hydrodynamic injection conditions were fixed at 50 mbar for 7 s, which ensured reproducible sample loading without capillary overloading or analyte leakage. Three critical independent variables were selected for the optimization phase: buffer concentration (15–50 mM), applied voltage (10–30 kV), and capillary temperature (25 °C–35 °C).

The D-optimal design generated an experimental design matrix composed of 13 runs (Table 2). In this matrix structure, each row represents an individual experimental run (or sample condition to be tested), while each column corresponds to a specific independent variable (buffer concentration, voltage, or temperature). This mathematically constructed matrix allows for the simultaneous evaluation of three key components via a second-order polynomial equation: the individual (linear) effect of each variable, the two-factor interactions (how variables influence each other), and the quadratic effects, which describe the curvature of the response surface.

Concomitant increases in both factors generated elevated currents, inducing Joule heating that compromised baseline stability and resolution. Conversely, excessively low buffer concentrations and voltages resulted in unstable current profiles and poor migration time reproducibility. These findings are consistent with electrophoretic theory, since increasing ionic strength simultaneously increases current intensity and heat generation, ultimately reducing separation efficiency.

The DoE model identified an optimal intermediate operating region, providing the ideal compromise between peak resolution, total analysis time, current stability, and overall method robustness. To thoroughly evaluate the significance of the experimental variables and their cross-effects, a comprehensive Analysis of Variance (ANOVA) was executed. The quadratic response surface model demonstrated exceptional statistical significance (F = 100.41, p = 0.0002), indicating that the selected mathematical framework accurately describes the electrophoretic behavior. The predictive capability and goodness-of-fit were confirmed by a high coefficient of determination (*R* ^2^ = 0.9951), proving that 99.5% of the observed variability in peak resolution capacity is successfully accounted for by the model. Crucially, the model allocated 9 degrees of freedom to the regression terms and 4 residual degrees of freedom for the error estimation, which provided adequate statistical power to map the experimental domain without overfitting.

The individual regression coefficients, standard errors, and corresponding *p*-values are detailed in Table 3. The linear terms for buffer concentration, applied voltage, and capillary temperature were all found to be highly significant (p < 0.01). Specifically, the negative quadratic coefficients for both buffer concentration (β_11_ = −0.0045, p = 0.0000) and voltage (β_22_ = −0.0122, p = 0.0005) mathematically confirm a concave curvature of the response surface, validating the presence of an optimal operating plateau at intermediate values within the explored domain. Furthermore, a highly significant negative interaction term was observed between buffer concentration and voltage (β_12_ = −0.0028, p = 0.0007).

**TABLE 3:** Regression coefficients, standard errors, and corresponding *p*-values for the second-order polynomial model derived from the 13-run D-optimal experimental design.

Factor	Coefficient	Standard error (SE)	p-value	Significance
Intercept (β_0_)	22.509	0.2520	0.0009	Highly significant
Buffer (β_1_)	+0.3591	0.0410	0.0010	Highly significant
Voltage (β_2_)	+0.5704	0.0540	0.0005	Highly significant
Temperature (β_3_)	−0.1045	0.0068	0.0001	Highly significant
Buffer^2^ (β_11_)	−0.0045	0.0002	0.0000	Highly significant
Voltage^2^ (β_22_)	−0.0122	0.0012	0.0005	Highly significant
Buffer*Voltage (β_12_)	−0.0028	0.0003	0.0007	Highly significant
Buffer *Temperature (β_13_)	−0.0002	0.0001	0.1161	Not significant
Voltage*Temperature (β_23_)	+0.0002	0.0002	0.3739	Not significant

This negative cross-effect provides mathematical evidence that simultaneous increases in ionic strength and electric field generate destructive Joule heating, which severely disrupts baseline stability and peak capacity. Conversely, the cross-terms involving temperature (β_13_ and β_23_) exhibited non-significant *p*-values (p > 0.05), indicating that the linear effect of temperature operates independently of the other operational parameters within the 25 °C–35 °C range.

**FIGURE 3:**
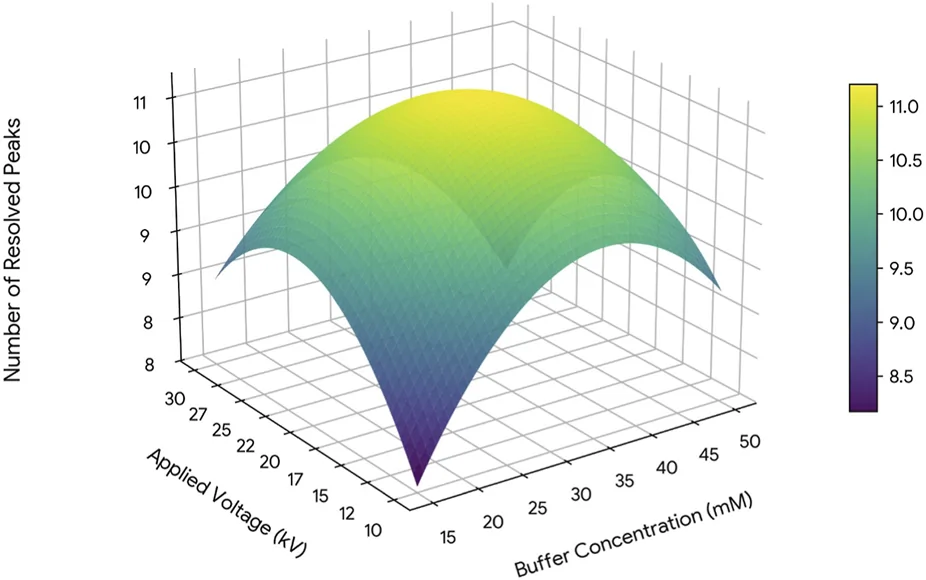
Response surface plots obtained from the D-optimal design for CE-DAD method optimization, illustrating the effects and interactions of buffer concentration (mM), applied voltage (kV), and capillary temperature (°C) on the analytical response. The colour gradient represents the magnitude of the predicted response, with blue/green areas corresponding to lower response values and yellow/red areas indicating progressively higher response values.

The relationships between the operational independent variables and the analytical responses were visually evaluated using three-dimensional response surface plots derived from the second-order model equation. Figure 3 illustrates the response surface generated for the number of resolved peaks as a function of buffer concentration and applied voltage, while holding the capillary temperature constant at its optimal setting of 25 °C. The topographical curvature of the surface demonstrates a pronounced concave profile, confirming that intermediate conditions represent the ideal experimental region to maximize peak capacity.

As observed in the plot, a maximum response plateau (Y = 11) successfully resolved peaks) is achieved in the central domain, specifically spanning a buffer concentration between 30 and 35 mM and an applied voltage from 18 to 22 kV. Below these values, the separation efficiency rapidly declines due to a weak electric field and insufficient ionic strength, which fail to selectively resolve the structurally similar aromatic amines. Conversely, when both buffer concentration and voltage are shifted toward their maximum limits (50 mM and 30 kV, respectively), a drastic collapse in the number of resolved peaks is observed. This behavior provides clear evidence of the negative interaction effect (β_12_ = −0.0028, p = 0.0007) discussed in the regression analysis, where excessive current generation triggers destructive Joule heating, baseline noise, and consequent peak co-elution.

To provide a comprehensive overview of the system behavior at higher thermal stress, the response surface model was also simulated at 35 °C (Supplementary Figure S1). At this elevated temperature, the global response surface shifts downward, narrowing the window of complete separation and verifying that lower operational temperatures (25 °C) are mandatory to preserve baseline stability and peak symmetry. Finally, the entire volume of the D-optimal design framework is mapped in Supplementary Figure S2, which displays the 3D experimental domain with all three operational factors on the physical axes, using a continuous four-dimensional color gradient to track the peak resolution profiles. Taken together, these response surfaces mathematically and visually justify that the final refined experimental conditions (33 mM buffer, 18 kV potential, and 25 °C capillary temperature) offer the most robust, sustainable, and reliable compromise to achieve full baseline separation of all eleven target restricted analytes.

The final optimized conditions are summarized in Table 4.

**TABLE 4:** Optimized CE-DAD operating conditions used for the determination of the target regulated aromatic amines in textile samples.

Buffer	Phosphoric acid pH 2.5
Buffer concentration	33 mM
Potential	18 KV
Current	42 Μa
Temperature	25 °C
Capillary	Silica 50 µm ID, 40 cm LEF
Electro-osmotic flow	—
DAD absorption wavelengths	214, 230, 250, 320 nm
Injection mode (PRESSURE)	50 psi
Injection mode (TIME)	7 s

**FIGURE 4:**
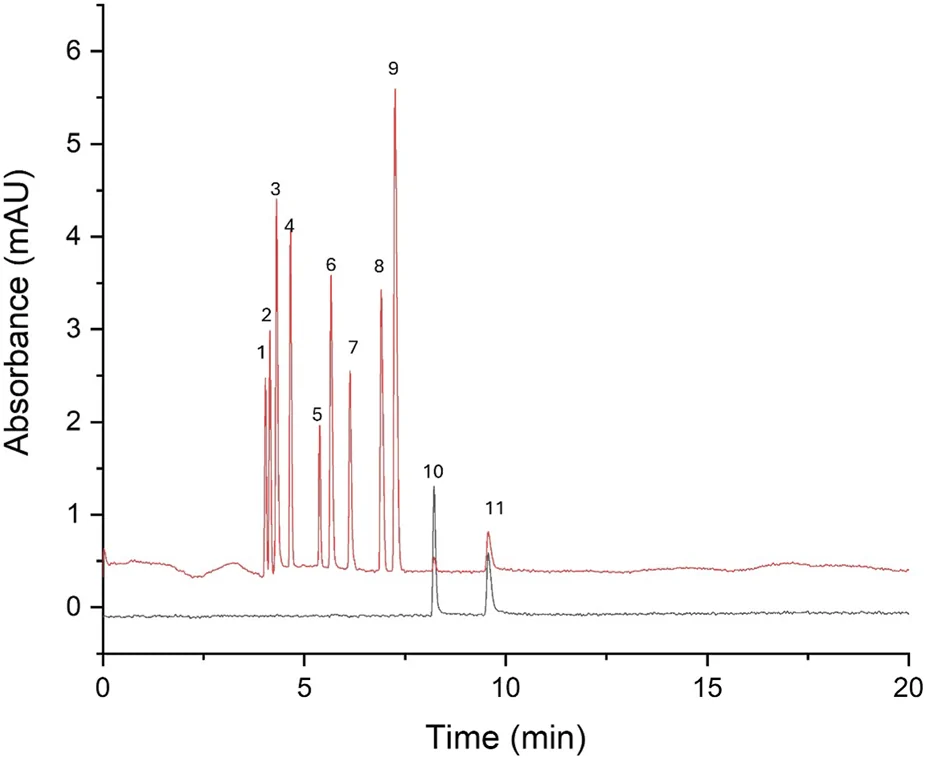
Electropherogram of a standard mixture containing the eleven-target regulated aromatic amines at a concentration of 5 μg mL^−1^ (equivalent to 3.3 μg g^−1^), obtained under the optimized CE-DAD conditions reported in Table 4. The electropherograms recorded at 214 nm and 320 nm are shown as red and dark traces, respectively. The peaks are assigned as follows: 1) benzidine (4.04 min); 2) o-toluidine (4.15 min); 3) 4,4′-diaminodiphenylmethane (4.30 min); 4) p-cresidine (4.66 min); 5) 4-chloro-o-toluidine (5.30 min); 6) 4-chloroaniline (5.70 min); 7) 4,4′-methylenedi-o-toluidine (6.16 min); 8) 4,4′-thiodianiline (6.90 min); 9) 4-aminobiphenyl (7.30 min); 10) o-aminoazotoluene (8.25 min); and 11) p-phenylazoaniline (9.60 min).

Under the optimized electrophoretic conditions, the multistandard solution containing the target aromatic amines was analyzed. All eleven analytes were successfully detected and baseline-resolved, with sharp, well-defined, and symmetrical peaks. The results demonstrated satisfactory selectivity and high reproducibility of the developed CE-DAD method, with migration times ranging from 4.0 to 10.0 min. Detection was carried out from 214 to 350 nm, selected according to the maximum UV absorbance of the individual analytes. The corresponding electropherogram is shown in Figure 4. The identification in the real sample migration was based on migration time, supported by multiple, complementary criteria, DAD spectral information, peak purity assessment, and standard-addition experiments performed on commercial textile samples. Therefore, although MS-based confirmation could provide additional structural information, the combination of migration time, DAD spectral matching, peak purity, and standard addition provides a robust basis for the identification of the investigated aromatic amines by CE-DAD in the analysed textile samples.

### 3.2 Method validation results

The analytical performance of the optimized CE–DAD method was evaluated in terms of selectivity, linearity, precision, matrix effect, DL/QL and extraction efficiency. Under the selected acidic electrophoretic conditions (pH 2.5), aromatic amines were present predominantly in their protonated form, resulting in selective electrophoretic migration and minimizing potential interferences from textile matrix components. The selectivity was confirmed by analysing blank textile extracts spiked after extraction, which showed no interfering signals at migration times of the target analytes. Matrix effect was negligible since matrix matched calibration curves showed slopes comparable to those obtained for standard solutions (ME% <10%). Therefore, solvent-based calibration was considered suitable for quantitative analysis.

All investigated analytes showed satisfactory, linear responses over the selected concentration ranges, with coefficients of determination (*R* ^2^) ranging from 0.9909 to 0.9989. Evaluation of the regression parameters and their 95% confidence intervals, together with the residual distributions, supported the adequacy of the linear regression models over the investigated analytical ranges. The regression equations, slopes, intercepts, corresponding 95% confidence intervals, and *R* ^2^ values are reported in Supplementary Table S1, while the residual plots are provided in Supplementary Figure S3.

The developed method exhibited good sensitivity, with DL values ranging from 0.2 to 0.6 μg mL^−1^ (corresponding to 0.1–0.4 μg/g) and QLvalues between 0.6 and 1.8 μg mL^−1^ (corresponding to 0.4–1.2 μg/g), confirming the suitability of CE–DAD for detecting aromatic amines at concentrations below the regulatory threshold. The QL achieved by the proposed CE–DAD method are comparable with the values reported for LC–MS/MS methods by Kämpfer et al. (2019), with limits of quantification ranging from 1 to 10 mg kg^−1^ for 58 aromatic amines and Tölgyesy and Sharma (2020) between 0.2 and 1 mg kg^−1^.

Method precision was evaluated through repeatability and intermediate precision experiments. Intra-day precision values ranged from 1 to 12% RSD, whereas intermediate precision varied between 2 and 14% RSD. The maximum RSD values represent the least favourable conditions. The observed precision was considered acceptable for the intended quantitative application, taking into account the complexity of the textile matrix and the complete analytical procedure, including extraction, preconcentration, and CE-DAD determination.

Extraction performance was evaluated through recovery experiments performed at the REACH regulatory concentration level (30 mg kg^−1^). Recovery was evaluated at a single concentration level (30 mg kg^−1^), which was selected because it corresponds to the applicable regulatory limit and is therefore particularly relevant to the intended application of the method. Values ranged from 90% to 105%, with RSD values below 8%, indicating efficient extraction of the target aromatic amines from textile matrices and good reproducibility of the complete analytical workflow. Alternative extraction and purification approaches, including microextraction by packed sorbent (MEPS) and salting-out-assisted liquid–liquid extraction (SALLE), have also been reported for the determination of aromatic amines in textiles. In that study, SALLE provided higher recoveries than MEPS. For the aromatic amines common to both studies, the recoveries obtained with the present PSE-based method were comparable to, or higher than, those achieved using SALLE (del Nogal Sánchez et al., 2014). Regarding robustness, no significant changes in migration times, peak areas, or critical resolution values were observed upon deliberate variation of the selected experimental parameters, confirming the robustness of the optimized CE–DAD method. The comprehensive validation results are reported in Table 5.

**TABLE 5:** Summary of the analytical validation parameters, their corresponding acceptance criteria, and the results obtained for the proposed CE-DAD method.

Parameter	Acceptance criterion	Result
Selectivity	Absence of interfering peaks	Confirmed
Stability	Variation after 24 h at 4 °C	Confirmed
Linearity	*R* ^2^ ≥ 0.99	0.995–0.999
DL	S/N ≈ 3	0.2–0.6 μg mL^−1^ (0.1–0.4 μg/g)
QL	S/N ≈ 10	0.6–1.8 μg mL^-1^ (0.4–1.2 μg/g)
Repeatability	RSD	1–12
Intermediate precision	RSD	2–14
Recovery	30 mg kg^−1^ spike level	90%–105% (RSD≤ 8)
Matrix effect	±10% negligible; ±10%–30% moderate	<10%
Robustness	No significant changes after deliberating variations	Confirmed

### 3.3 Analysis of commercial textile samples and assessment of method applicability

**FIGURE 5:**
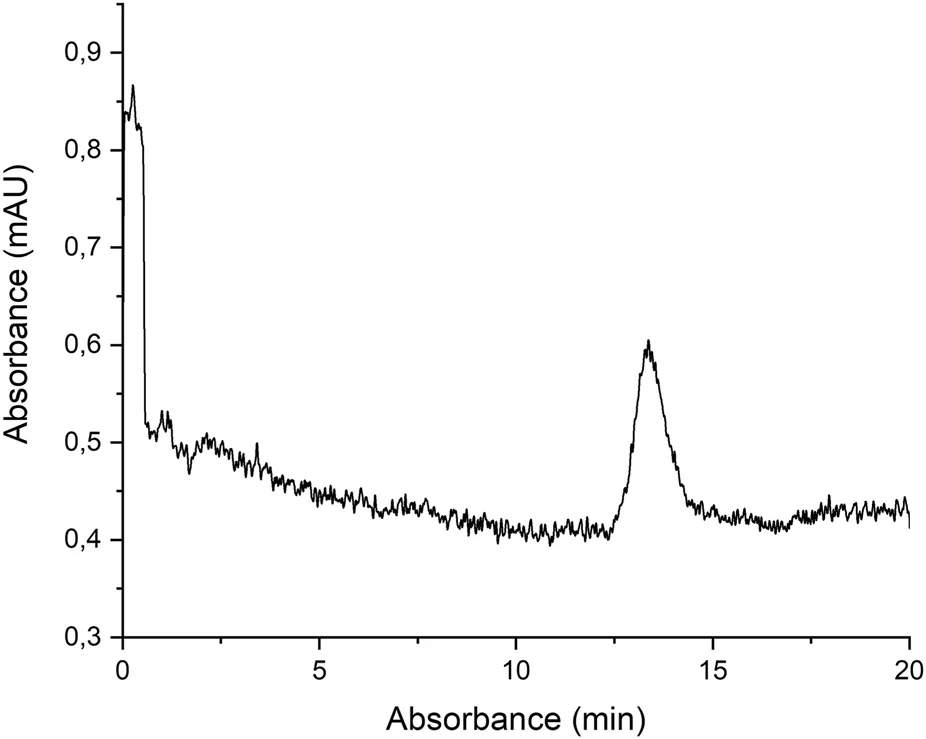
Electropherogram of the blank textile extract (Sample1) analysed under the optimized CE–DAD conditions, reported in Table 4. No interfering peaks were observed at the migration times of the target aromatic regulated amines.

**FIGURE 6:**
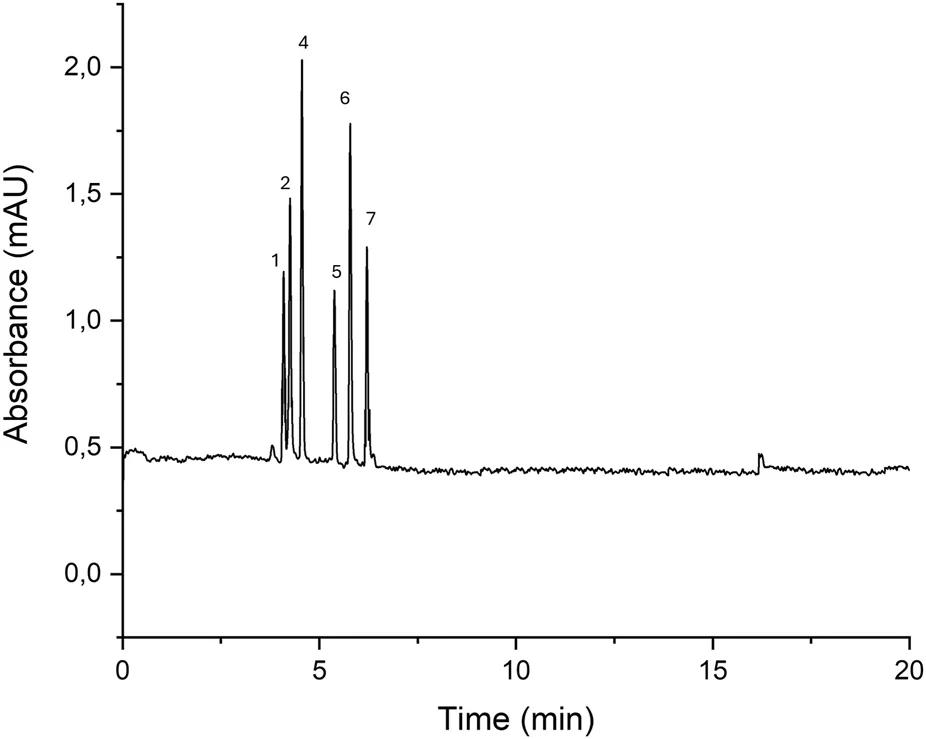
Electropherogram of the fortified blank textile extract (Sample 1) spiked with the target regulated aromatic amines at a concentration of 2.5 mg kg^−1^ and analyzed under the optimized CE-DAD conditions reported in Table 4. The electropherogram demonstrates the detection and separation of the spiked target analytes in the textile matrix under the optimized analytical conditions.

**FIGURE 7:**
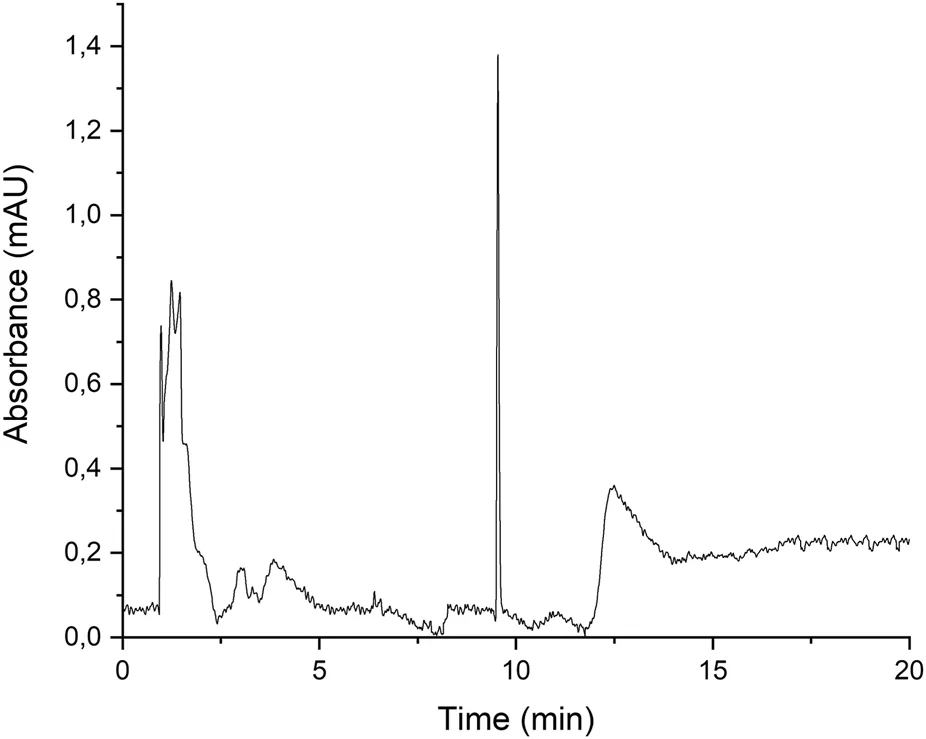
Electropherogram of textile sample 2 showing the detection of p-phenylazoaniline under the optimized CE-DAD conditions. Detection was performed at 320 nm.

**FIGURE 8:**
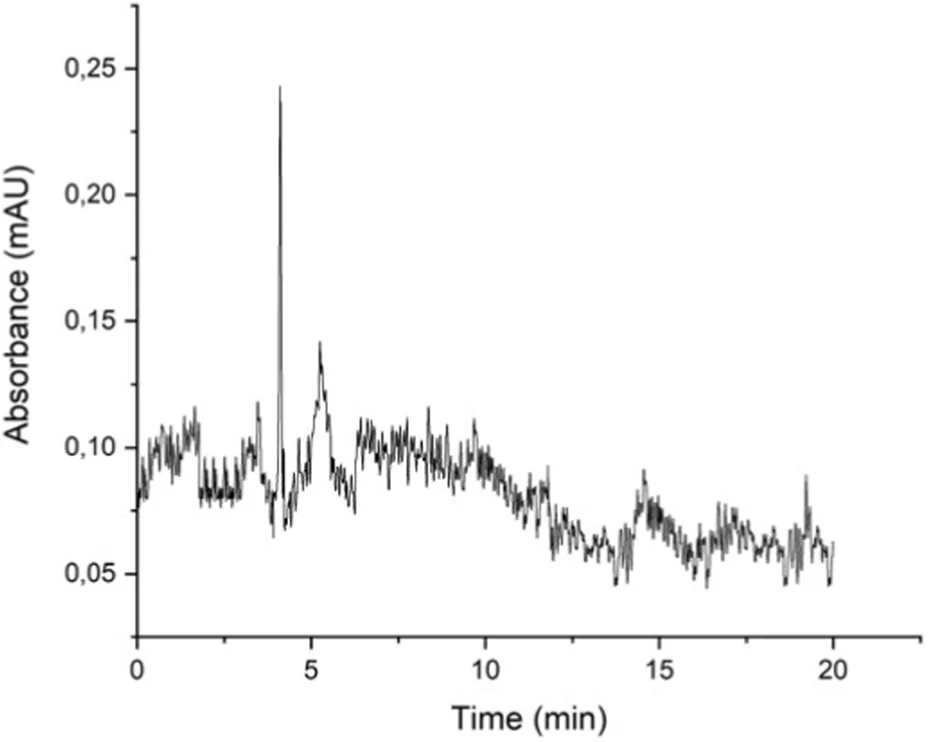
Electropherogram of textile sample 3 showing the detection of o-toluidine under the optimized CE-DAD conditions.

To evaluate the applicability of the proposed CE–DAD method to real textile matrices, a blank white textile sample, a fortified textile blank sample, and four commercial textile samples were analyzed. Sample 1 was a white textile selected as the blank matrix for the evaluation of selectivity, recovery, and matrix effects. The absence of visible coloration indicated that the textile was not intentionally dyed; however, this characteristic alone cannot be considered sufficient evidence for the absence of dyes or target amines. The sample was therefore considered a blank matrix based on its white appearance and the absence of detectable target analytes by the CE-DAD method. No independent orthogonal technique was used to establish its analyte-free status. Representative electropherograms are shown in Figures 5–8.

The electropherogram of the blank textile sample (Figure 5) showed no detectable peaks at the migration times of the target aromatic amines, demonstrating the absence of significant matrix interferences.

This result confirms the good selectivity of the proposed analytical method and indicates that the textile matrix does not compromise analyte identification or quantification under the optimized electrophoretic conditions.

To further evaluate method performance in the textile matrix, a blank textile sample was fortified with selected aromatic amines at a concentration of 2.5 mg kg^−1^ prior to analysis. Well-defined and symmetrical peaks were obtained for all analytes, with migration times consistent with those of the corresponding standard solutions. The method provided complete separation within a few minutes while maintaining satisfactory resolution and sensitivity. Since the fortification level corresponds to approximately one-twelfth of the REACH limit (30 mg kg^−1^), these results demonstrate that the proposed CE–DAD method is suitable for the detection of aromatic amines well below the regulatory threshold (Figure 6).

The six spiked aromatic amines were detected at their expected migration times, confirming analyte identification and the absence of significant matrix interferences.

Among the four commercial textile samples analyzed, only two contained detectable levels of aromatic amines. Specifically, Figure 7 shows the presence of p-phenylazoaniline in a yellow textile sample 2, whereas Figure 8 shows the detection of o-toluidine in sample 3 at concentrations of 15 mg kg^−1^, and below the QL level, respectively. In all cases, the measured concentrations were well below the regulatory limit established by the REACH Regulation, indicating compliance of the analyzed products with current European legislation.

The detection of p-phenylazoaniline and o-toluidine suggests the possible presence of azo dye-related structures within the sample. However, direct detection of these compounds does not permit identification of the parent dye(s), since they may represent dyes, synthetic intermediates, impurities, or degradation products, and several structurally distinct azo dyes may be associated with the same aromatic amines.

The aromatic amines detected in the samples investigated are among those frequently reported in textile products. Crettaz et al. (2020) reported the occurrence of 4-aminoazobenzene in a black children’s T-shirt at a concentration of 652 mg kg^−1^. The compound was confirmed according to ISO 14362-3 after the initial detection of aniline and p-phenylenediamine using the ISO 14362-1 procedure, demonstrating that significant levels of this aromatic amine may occur in textile products containing specific azo dyes. Tahara et al. (2024) included p-phenylazoaniline among the target analytes in their GC–MS method for textiles and leather products, highlighting its relevance as a regulated aromatic amine associated with specific azo dye structures.

In a survey by Kawakami et al. (2012) based on a GC–MS method, o-toluidine was detected at low level (below 1.0 μg/g) in textiles, but it was determined at a high concentration in one leather article, exceeding the REACH threshold of 30 mg/kg. In a previous paper o-toluidine was detected in one cotton placemat textile sample at a concentration of 6.8 μg/g (Kawakami et al., 2010.) More recently Kawakami et al. (2022) detected o-toluidine as an impurity in 3 out of 38 synthetic organic colorants at concentrations ranging from 1.0 to 3.4 mg/kg. These levels are below the REACH Annex XVII entry limit of 30 mg/kg applicable to aromatic amines released from azo dyes in finished textile and leather articles.

The consistency of the present findings with previously published data (Tahara et al., 2024; Kawakami et al., 2010; Kawakami et al., 2012; Kawakami et al., 2022) supports the reliability of the proposed CE–DAD method and demonstrates its potential as a rapid and environmentally sustainable approach for the monitoring of some regulated aromatic amines in textile products. Although ISO 14362-1:2017 recommends chromatographic techniques, particularly GC–MS, as the reference method for the confirmatory determination of aromatic amines released from azo colorants in textiles, LC–MS/MS has also been proposed for confirmatory and quantitative analysis (Tölgyesi and Sharma, 2020). Despite the lack of structural information provided by DAD compared with mass spectrometric detection, the optimized CE–DAD method achieved complete baseline separation of the investigated analytes, with no significant co-elution from textile matrix components. Consequently, the proposed method is well suited for screening and quantitative determination of the selected aromatic amines, offering reduced solvent consumption, shorter analysis times, and lower environmental impact. However, confirmatory analysis of positive or ambiguous samples may still require MS-based techniques.

### 3.4 Greenness and applicability assessment

The environmental impact and practical applicability of the developed analytical procedure were evaluated using the complementary AGREE and BAGI metrics. The AGREE assessment provided a quantitative measure of the compliance of the entire analytical workflow with the twelve principles of Green Analytical Chemistry. Default weights (2) were applied, except for the criteria related to the integration of operations (criterion 4) and productivity (criterion 8), whose weights were increased by two. The limited number of sample-preparation steps justified the higher weight assigned to criterion 4, while the automated ASE extraction combined with the short CE analysis time supported the increased weight for criterion 8.

**FIGURE 9:**
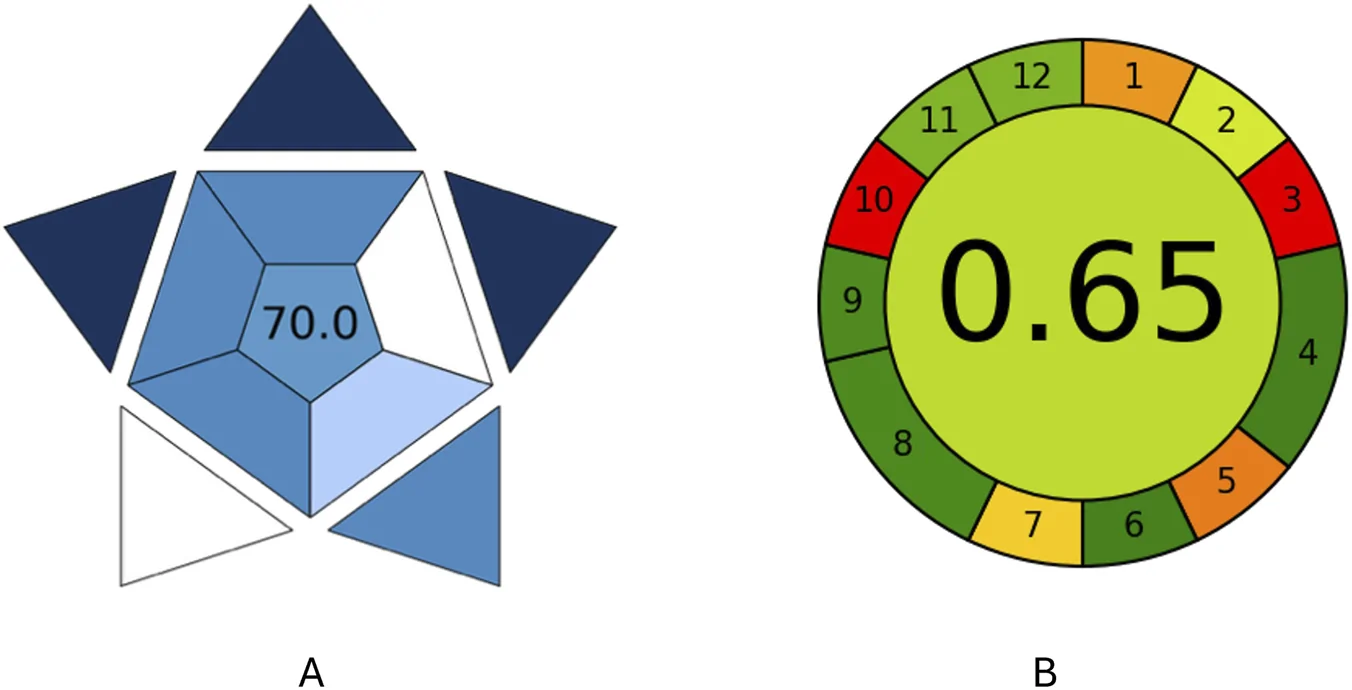
Greenness assessment of the proposed CE-DAD analytical method using **(A)** the BAGI and **(B)** the AGREE metric.

Several features of the proposed CE-DAD method contributed positively to its environmental profile, including the very small injection volume, short electrophoretic analysis time, low energy consumption, simultaneous determination of eleven target analytes, and limited reagent consumption during electrophoretic separation. In addition, the use of a multivariate DoE strategy reduced the number of experiments required for method optimization, thereby minimizing reagent consumption and analytical waste generation. The main limitations identified by AGREE were associated with the extraction procedure. The use of methanol containing 0.1% NH_4_OH penalized criteria related to solvent volume (criterion 7), the non-renewable nature of the solvent (criterion 10), and reagent safety due to methanol toxicity (criterion 11). Consequently, sample preparation represented the stage with the greatest potential environmental impact. An additional penalty was observed for criterion 3, related to the positioning of the analytical device. Overall, the AGREE score was 0.65 (Figure 9B), indicating a favorable environmental profile and good compliance with the principles of Green Analytical Chemistry.

The practical applicability of the method was in parallel evaluated using BAGI. A score of 70.0 (Figure 9A) was obtained, exceeding the threshold of 60 recommended for classification as a practically feasible analytical method. The favorable score was mainly attributable to the use of common reagents and materials, the small sample amount required, satisfactory analytical throughput, and the simultaneous determination of multiple analytes. The main limitations concerned the sample preparation procedure, which involves multiple steps and requires preconcentration, as well as the limited possibility of processing samples in parallel. Overall, the BAGI score indicates a good level of practical applicability and supports the suitability of the method for the selected applications. Further improvements could be achieved by simplifying the sample-preparation workflow and reducing or eliminating the preconcentration steps. Taken together, the AGREE and BAGI results indicate a favorable balance between environmental sustainability and practical applicability, while also highlighting sample preparation as the main area for future optimization (Nowak et al., 2021).

## 4 Conclusion

A green, rapid, and reliable CE–DAD method was successfully developed for the determination of eleven REACH-regulated aromatic amines in textile samples. The application of a multivariate Design of Experiments (DoE) approach enabled the efficient optimization of the electrophoretic conditions while reducing the number of experimental trials, reagent consumption, and analytical waste, in accordance with the principles of Green Analytical Chemistry. The optimized method exhibited satisfactory analytical performance, including good linearity, low detection limits, adequate precision, and accurate recoveries, demonstrating its suitability for the determination of aromatic amines in complex textile matrices.

The application of the proposed workflow to real textile samples following PSE extraction confirmed its capability to detect regulated aromatic amines at low concentration levels and demonstrated its applicability for routine analysis of the selected amines. Compared with conventional chromatographic methods, the proposed CE–DAD approach requires significantly lower solvent consumption, reduces operational costs and environmental impact, and provides rapid analysis, making it an attractive complementary tool for the screening and monitoring of hazardous aromatic amines in textile products. The integration of DoE for systematic method optimization together with the assessment for evaluating analytical greenness highlights the robustness and sustainability of the proposed methodology. In particular, the complementary AGREE and BAGI assessments provided an overall favorable evaluation of the method, with an AGREE score of 0.65 and a BAGI score of 70.0, the latter exceeding the threshold of 60 for practical feasibility. These results confirm a favorable balance between environmental sustainability and practical applicability and support the suitability of the proposed CE–DAD method for routine analytical applications. The positive environmental profile was mainly associated with the low sample and reagent consumption, small injection volume, short analysis time, low energy demand, simultaneous determination of eleven analytes, and the reduction of experimental waste achieved through the DoE strategy. Conversely, both assessments identified sample preparation, particularly the methanolic extraction and the preconcentration and multiple processing steps, as the main area limiting the overall greenness and practical applicability of the workflow. Overall, this study demonstrates that capillary electrophoresis can provide reliable analytical performance while complying with Green Analytical Chemistry principles, offering a practical and accessible alternative for routine quality control, market surveillance, and consumer protection. Future improvements should therefore focus on simplifying the sample-preparation procedure, reducing or eliminating preconcentration steps, decreasing solvent consumption and toxicity, and increasing the possibility of parallel sample processing. Future work will focus on extending the method to additional regulated aromatic amines and broader classes of textile materials.

## Data Availability

The original contributions presented in the study are included in the article/Supplementary Material, further inquiries can be directed to the corresponding author.
