# Supplementary material for: Green CE–DAD determination of regulated aromatic amines in textiles through DoE optimization
Source: Front Chem. 2026 Sep 9;14:1952218. doi: 10.3389/fchem.2026.1952218 (PMC13597927; doi:10.3389/fchem.2026.1952218)
Supplement: Supplementary file 1 [file DataSheet1.docx]

Supplementary Material

**Green CE–DAD Determination of Regulated Aromatic Amines in Textiles through DoE Optimization**

**Giulia Simonetti^1^, Francesca Buiarelli^1*^, Veronica Mari^1^, Sara Astolfi^1^, Fabio Candiano^1^, Eugenio Sandrucci^2^, Carmela Riccardi^3^, Donatella Pomata^3^, Patrizia Di Filippo^3^**

^1^Department of Chemistry “Sapienza” University of Rome P. le Aldo Moro, 5 -00185 Rome-Italy

^2^Wolfson School of Mechanical, Electrical and Manufacturing Engineering University of Loughborough Ashby Road, Loughborough LE11 3TU, UK

^3^ Inail DIT-Via Roberto Ferruzzi, 38-00143 Rome, Italy

corresponding author Francesca Buiarelli e-mail: francesca.buiarelli@uniroma.it

**Table S1**. Linear regression parameters for the calibration curves of the 11 aromatic amines over the investigated analytical ranges. Regression equations, slopes and intercepts with their corresponding 95% confidence intervals (CIs), and coefficients of determination (R²) are reported.

| **Analyte** | **Regression equation** | **Slope (95% CI)** | **Intercept (95% CI)** | **R²** |
| --- | --- | --- | --- | --- |
| 4,4′-Methylenedi-o-toluidine | y = 0.9858x + 0.8982 | 0.9858 (0.8545–1.1171) | 0.8982 (0.1603–1.6361) | 0.9909 |
| Benzidine | y = 1.4170x + 0.3729 | 1.4170 (1.2768–1.5571) | 0.3729 (−0.4146–1.1605) | 0.9949 |
| 4,4′-Diaminodiphenylmethane | y = 1.5497x + 0.3203 | 1.5497 (1.4608–1.6385) | 0.3203 (−0.1789–0.8196) | 0.9983 |
| 4,4′-Thiodianiline | y = 0,9416x − 0,5181 | 0,9416 (0,7943–1,0889) | −0,5181 (−1,4238–0,3876) | 0.9928 |
| 4-Chloroaniline | y = 0,5687x + 0,0274 | 0,5687 (0,4683–0,6690) | 0,0274 (−0,5897–0,6446) | 0.9909 |
| 4-chloro-o-toluidine | y = 0,9875x + 0,0009 | 0,9875 (0,8723–1,1027) | 0,0009 (−0,6462–0,6480) | 0.993 |
| p-Cresidine (6-methoxy-m-toluidine) | y = 0.8065x + 0.2415 | 0.8065 (0.6791–0.9339) | 0.2415 (−0.5418–1.0248) | 0.9927 |
| o-Toluidine (2-aminotoluene) | y = 1.3652x + 0.0545 | 1.3652 (1.1969–1.5336) | 0.0545 (−0.8915–1.0005) | 0.9922 |
| 4-Aminobiphenyl (Biphenyl-4-ylamine, 4-aminobiphenyl xenylamine) | y = 2.7386x + 2.3841 | 2.7386 (2.4518–3.0255) | 2.3841 (0.7723–3.9959) | 0.9943 |
| p-Phenylazoaniline (4-Aminoazobenzene) | y = 1.2441x + 0.1514 | 1.2441 (1.0961–1.3921) | 0.1514 (−0.6804–0.9833) | 0.9927 |
| o-Aminoazotoluene | y = 1.0069x + 0.1913 | 1.0069 (0.9602–1.0536) | 0.1913 (−0.0709–0.4536) | 0.9989 |

**Figure S1.** Response surface plot showing the effects of buffer concentration (mM) and applied voltage (kV) on the number of resolved peaks (capillary temperature held at 35 °C).

**Figure S2.** 3D representation of the experimental domain (buffer, voltage, and temperature) with the colour gradient mapping the number of resolved peaks.

|  |  |
| --- | --- |
|  |  |
|  |  |
|  |  |
|  |  |
|  |  |

**Figure S3.** Residual plots for the calibration curves of the 11 aromatic amines. Residuals were calculated as the difference between the observed and predicted responses and plotted against concentration.
